# Ultrasound Image Temperature Monitoring Based on a Temporal-Informed Neural Network

**DOI:** 10.3390/s24154934

**Published:** 2024-07-30

**Authors:** Yuxiang Han, Yongxing Du, Limin He, Xianwei Meng, Minchao Li, Fujun Cao

**Affiliations:** 1 School of Digital and Intelligence Industry, Inner Mongolia University of Science & Technology, Baotou 014000, China; yuxianghan@stu.imust.edu.cn (Y.H.);; 2 School of Science, Inner Mongolia University of Science & Technology, Baotou 014000, China; 3 Laboratory of Controllable Preparation and Application of Nanomaterials, Technical Institute of Physics and Chemistry, Chinese Academy of Sciences, Beijing 100190, China

**Keywords:** microwave hyperthermia, ultrasound, non-invasive temperature monitoring, neural networks

## Abstract

Real-time and accurate temperature monitoring during microwave hyperthermia (MH) remains a critical challenge for ensuring treatment efficacy and patient safety. This study presents a novel approach to simulate real MH and precisely determine the temperature of the target region within biological tissues using a temporal-informed neural network. We conducted MH experiments on 30 sets of phantoms and 10 sets of ex vivo pork tissues. We proposed a novel perspective: the evolving tissue responses to continuous electromagnetic radiation stimulation are a joint evolution in temporal and spatial dimensions. Our model leverages TimesNet to extract periodic features and Cloblock to capture global information relevance in two-dimensional periodic vectors from ultrasound images. By assimilating more ultrasound temporal data, our model improves temperature-estimation accuracy. In the temperature range 25–65 °C, our neural network achieved temperature-estimation root mean squared errors of approximately 0.886 °C and 0.419 °C for fresh ex vivo pork tissue and phantoms, respectively. The proposed temporal-informed neural network has a modest parameter count, rendering it suitable for deployment on ultrasound mobile devices. Furthermore, it achieves temperature accuracy close to that prescribed by clinical standards, making it effective for non-destructive temperature monitoring during MH of biological tissues.

## 1. Introduction

Cancer incidence rates have been progressively increasing in recent years [1], necessitating ongoing research and technological advancements in tumor treatment methods. Microwave hyperthermia (MH) is a minimally invasive local therapeutic approach [2,3] often used alongside chemotherapy and radiotherapy. Depending on tissue heating levels, MH is categorized into mild hyperthermia and thermo-ablation. Mild hyperthermia maintains tissue temperatures within 41–45 °C and is more effective at stimulating the body immune system than thermo-ablation [4]. The temperature range for thermo-ablation is 46–60 °C, with the aim of inducing irreversible cellular damage and causing cellular denaturation [5].

During MH, real-time biological-tissue temperature monitoring is crucial for several reasons. First, it helps users determine whether the desired therapeutic temperature range is achieved. Second, it promptly alerts users when temperatures reach levels that could potentially cause thermal damage to biological tissues, thereby safeguarding the healthy tissues of patients.

Currently, several methods are utilized to monitor temperature in the treatment region, including the use of thermistors [6], thermocouples [7], and optical fiber temperature sensors [8]. However, these methods are invasive and may lead to tissue damage. Non-invasive methods such as magnetic resonance imaging [9] and computed tomography [10] also have drawbacks. Magnetic resonance imaging can be challenging for real-time tissue temperature monitoring, whereas computed tomography involves high radiation doses and equipment costs, which render them less suitable for non-destructive temperature monitoring during MH.

Conversely, ultrasound is widely used owing to its affordability, minimal harm to the body, and real-time capabilities for non-destructive temperature detection [11]. However, ultrasound temperature measurement has limitations in terms of detection accuracy, which restricts its clinical applications.

In contrast to temperature-measurement methods using ultrasound radiofrequency signals [12,13,14,15,16], temperature research based on ultrasound images is more universal because most ultrasound imaging devices do not directly acquire ultrasound radiofrequency signals; they produce ultrasound images. Therefore, research has focused on image analysis [17,18]. Changes in ultrasound B-mode images can be used to estimate temperature variations. The underlying assumption of this theory is that temperature changes cause alterations in the speed of sound propagation, affecting ultrasound reflection signals. These changes manifest as alterations in grayscale and texture structures on ultrasound images.

Teixeira et al. [19] demonstrated the feasibility of estimating temperature relations in bovine muscle tissue using therapeutic ultrasound as a heat source. They found a temperature-estimation error of below 0.5 °C when extrapolating temperature relations from one region to another, indicating a linear relationship between the average grayscale level (AVGL) and temperature. Alvarenga et al. [20,21] established the AVGL–temperature relationship in the range 37–46 °C using heating plates on phantom materials, with a ratio of 2.03 °C/AVGL. They also observed a correlation coefficient of 0.84 between temperature changes and two grayscale co-occurrence matrix (GLCM) parameters (correlation and entropy) extracted from images when using water bath heating on phantom materials. Based on previous studies, they [22] limited the maximum uncertainty of AVGL-estimated temperature from 35 °C to 41 °C using water bath heating to 0.68 °C. Rigueira et al. [23] identified the smallest possible regions in images where changes in grayscale pixel intensity were perceptible to human observers. Wang and Sheng [24] conducted warming experiments using water bath heating to reconstruct B-mode ultrasound images. They extracted texture features based on a grayscale histogram, GLCM, and grayscale gradient co-occurrence matrix and found that five texture feature parameters correlated with temperature with correlation coefficients exceeding 0.9.

However, the aforementioned studies have several limitations: they [15,19] estimate temperature from ultrasound images obtained during single heating experiments without testing different experimental groups; they [20,21,22,24] use heating plates or water bath heating instead of MH, which can introduce discrepancies compared with actual experiments; some studies [20,21,22] have a limited temperature-monitoring range, typically around 35–40 °C, thus failing to consider temperatures within the thermo-ablation range relevant to microwave treatment.

This study was conducted to address the aforementioned shortcomings. We summarize the contributions of our study as follows:

1. We designed MH heating experiments on biological tissues under ultrasound diagnostic equipment irradiation, controlling tissue temperature within the range 25–65 °C. During MH, we collected ultrasound B-mode images along with tissue temperature data in the range of 25–65 °C.
2. We suggested, through an analysis of experimental data, that ultrasound image feature parameters at different tissue temperatures can be regarded as sequences containing temporal information. These sequences exhibit different short heating periods within a complete heating period due to material composition variations. We validated this hypothesis by observing the frequency-domain plots of this sequence using fast Fourier transform (FFT).
3. We proposed a hybrid model for non-invasive ultrasound temperature monitoring in biological tissues, combining TimesNet [25] and the Cloblock module from Cloformer [26]. Our proposed temporal-information-based neural network outperformed single models or previous temperature-measurement methods in terms of model-parameter count and temperature accuracy.

The remainder of this paper is structured as follows. In the Materials and Methods section, we introduce the MH experiments and theoretical approaches used in this study, outlining the process of extracting ultrasound image parameters and constructing the neural-network model. The Results section presents the results and temperature-prediction images from the hyperthermia experiments. Thereafter, we discuss the experimental findings and draw conclusions.

## 2. Materials and Methods

### 2.1. Experimental Setup

**Figure 1:**
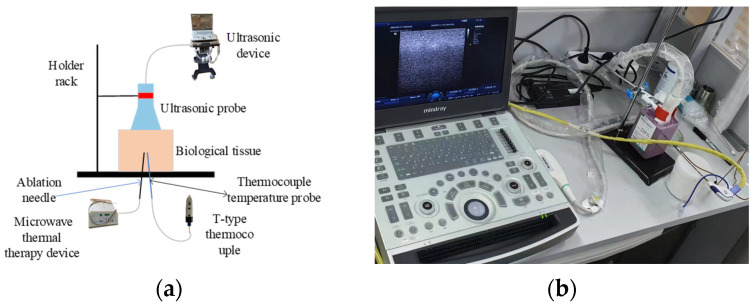
Experimental setup. (**a**) Schematic of the experimental system illustrating the positions of various experimental apparatus. (**b**) Overview of the experimental environment.

The MH experimental setup includes an MH device and temperature-measurement and ultrasound-detection equipment (Figure 1). The specific MH device used is the MH-IY model manufactured by Beijing Muheyu Electronics Co., Ltd. (Beijing, China), operating at a frequency of 2450 MHz for the ablation needle. Temperatures were measured using a T-type thermocouple WRNT-01 produced by Kapson Company (Croydon, UK), offering an effective measurement range of −200 °C to 260 °C, with an accuracy of 0.01 °C and a measurement frequency of once per second. Ultrasound was detected using the M9Vet portable ultrasound diagnostic instrument from Mindray Company, set to small organ mode with a sampling frequency of 17.25 MHz. Fresh ex vivo pork tissue (pork tissue structure and dielectric constant are similar to those of human tissue [27]) and phantom materials were used. The pork tissue was obtained from a pig slaughtered on the day of the experiment at a local supermarket. The tissue was cut into cubic pieces of approximately 6 cm in length, 4.5 cm in width, and 3 cm in height; 10 pieces were selected for the experiment. The phantom materials were prepared using AW Guy’s formulation [28], comprising TX-150, deionized water, polyethylene powder, sodium chloride, and a thermochromic material that changes color from purple to pink when the temperature exceeds 40 °C. After thorough mixing using an ultrasonic vibrator, the mixture was placed in a sterile culture medium to form a cubic structure measuring 5 cm in length, 5 cm in width, and 9 cm in height.

### 2.2. Experimental Procedure

The ex vivo pork tissue was positioned horizontally on the specimen stage. The MH probe and T-type thermocouple temperature probe were inserted in parallel in the central position of the pork tissue, maintaining a horizontal distance of approximately 5 mm between the tips of the two probes. The ultrasound probe was oriented orthogonally to the plane of irradiation, enabling clear observation of both probes on the display interface of the ultrasound-detection equipment. The MH device and temperature recording function of the thermocouple were simultaneously activated. The MH device was set to an output power of 5 W, with a heating duration of 8 min. This experimental procedure was applied to the 10 pieces of pork tissue under the same conditions, including ambient temperature, probe distance, and output power, to ensure the reproducibility of the experiments.

The same experimental procedure was applied to the phantom materials—30 heating experiments were performed. A constant initial room temperature was maintained before each experiment on the phantom materials to ensure uniform and controlled conditions throughout the experiments.

### 2.3. Data Processing

**Figure 2:**
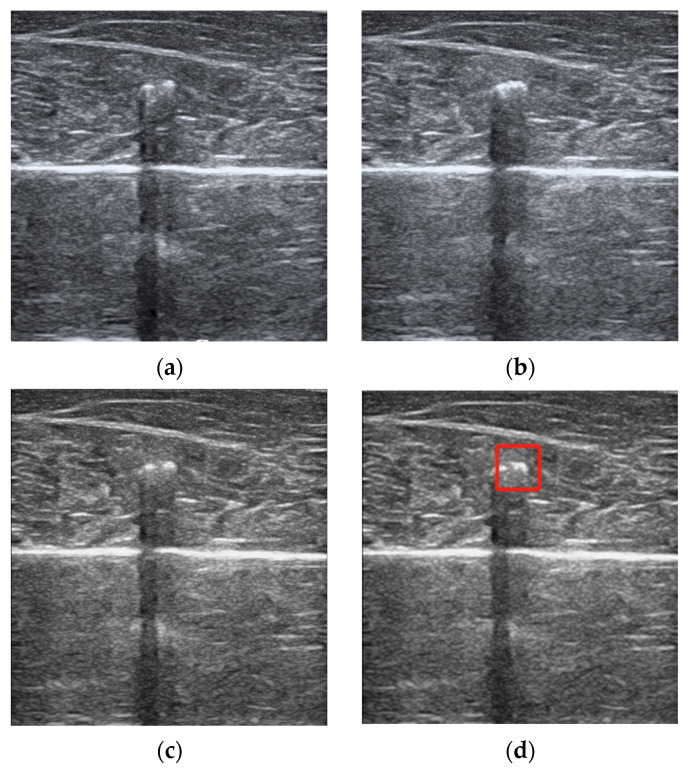
(**a**) ultrasound image before heating; (**b**) ultrasound image after heating; (**c**) image fused along the time dimension; (**d**) image after mean filtering.

The ultrasound video file, recorded in .AVI format with a duration of 8 min and a frame rate of 30 frames per second, was processed along with the corresponding temperature-probe-recorded heating curve. The images from each frame of the video file were captured, and the time dimensions of the ultrasound images were fused to align the image frequency with the temperature sampling frequency (Figure 2c). Subsequently, each image underwent mean filtering [29] to remove speckle noise. A region of interest measuring 64 × 64 pixels, centered around the temperature probe, was selected for further processing, as indicated by the red box in Figure 2d.

### 2.4. Ultrasound Image Feature Parameters

We employed the gray-level gradient co-occurrence matrix (GLGCM) method to extract texture features from the images. This method integrates edge-gradient information into the joint statistical distribution of spatial gray levels. In contrast to the conventional GLCM, GLGCM captures grayscale space information and incorporates edge-gradient information from the image [30]. Grayscale space represents the fundamental brightness variations in the image, reflecting changes in ultrasound echo intensity. Conversely, gradient space depicts edge information within the image. By combining these two information spaces, we can effectively capture texture variations in ultrasound images across different temperatures.

First, let f(i,j) denote the ultrasound grayscale image and g(i,j) represent the corresponding gradient image obtained through the gradient operator ∇. They are normalized according to Equations (1) and (2), where N and M represent the maximum number of gray levels after normalization for the grayscale image f(i,j) and the gradient image g(i,j) respectively. $f_{max}$ and $f_{max}$ are the maximum and minimum values of f(i,j), respectively. $g_{max}$ and $g_{min}$ are the maximum and minimum values of $g\left(i,j\right)$, respectively. $F\left(i,j\right)$ and $G\left(i,j\right)$ denote the normalized grayscale matrix and gradient matrix, respectively.

(1) $$F\left(i,j\right)=\left[\frac{f\left(i,j\right)}{f_{max}-f_{min}}\times N\right]+1$$

(2) $$G\left(i,j\right)=\left[\frac{g\left(i,j\right)}{g_{max}}\times M\right]+1$$

These equations illustrate the normalization process for both grayscale and gradient images, which is a crucial step in preparing the data for subsequent analysis.

GLGCM $\left\{h\left(x,y\right),x=0,1,2,\ldots ,N-1;y=0,1,2,\ldots ,M-1\right\}$, is defined as the number of elements with $F\left(i,j\right)=x$ and $G\left(i,j\right)=y$, $F\left(i,j\right)$ and $G\left(i,j\right)$ denote the normalized ultrasound image normalized grayscale matrix and gradient matrix respectively. The normalized GLGCM is expressed according to Equation (3):

(3) $$H\left(x,y\right)=\frac{h\left(x,y\right)}{\sum_{x=0}^{N-1}\sum_{y=0}^{M-1}h\left(x,y\right)}$$

Based on $H\left(x,y\right)$, 15 s order feature parameters can be extracted to describe the texture features of the image. Additionally, we introduced a feature parameter: gray-temperature gradient, denoted as $r_{X,T}$, which is calculated using the Pearson correlation coefficient with temperature, as shown in Equation (4):

(4) $$r_{X,T}=\frac{\mathrm{cov}\left(X,T\right)}{\sigma_{X}\sigma_{T}}$$

Here, X represents the second-order feature parameters; T represents the temperature corresponding to the current ultrasound image; cov⁡(X,T) denotes the covariance between X and T; $\sigma_{X}$ and $\sigma_{T}$ represent the standard deviations of X and T, respectively.

We selected the top six feature parameters with the highest Pearson correlation coefficients with temperature (the remaining correlation coefficients were all less than 0.5), which are defined in Table 1, where their respective Pearson correlation coefficients are presented.

Accordingly, gray-temperature grad represents the ratio of temperature variation to grayscale variation, where ${AVGL}_{t}$ and $T_{t}$ denote the grayscale mean value and corresponding temperature values at time t, respectively. ${AVGL}_{t+5}$ and $T_{t+5}$ denote the grayscale mean value and corresponding temperature value at time t+5, respectively.

Following the described formulas, feature parameters were extracted from the images obtained during the experiments. Subsequently, a dataset that correlates these feature parameters with temperature for both pork tissue and phantom models was constructed. This dataset was instrumental in establishing the relationship between the extracted image features and the corresponding temperatures, which facilitated the development and validation of temperature-estimation models based on ultrasound image analysis.

### 2.5. Model Design

The feature-parameter sequence at consecutive time points under fixed microwave radiation power (5 W) showed temporal dependence. The feature-parameter sequences corresponding to ultrasound images did not conform to the assumption of independent and identically distributed data. The image feature parameters at that moment were influenced by the image feature parameters from previous moments, indicating temporal dependence. Moreover, the semantic information provided by a single image was insufficient for accurate temperature estimation. The content of a single image was inadequate to fully represent all the temperature information at a given moment, as the heating information is embedded within a sequence that includes time-related information about the heating period. Previous attempts [31] to model ultrasound multi-feature-parameter sequences using linear or nonlinear fitting encountered difficulties: conventional convolutional networks designed for time-series data employing one-dimensional convolutional kernels struggle to effectively capture transformations across multiple time steps, limiting their ability to model long-term, multi-period sequences.

The MH principle involves the absorption of electromagnetic energy by molecules within biological tissues, which is then converted into molecular kinetic energy [32]. This process leads to heat generation owing to friction between molecules, increasing tissue temperature. Inspired by concepts from food engineering, where proteins, fats [33,34], and carbohydrates in biological tissues exhibit different dielectric constants, MH relies on dielectric heating. When exposed to an alternating microwave electromagnetic field, dielectric heating reactions occur [32,35].

The pork tissue used in this study consists of proteins, fats, carbohydrates, water, and other components. We hypothesize that different components within the tissue, such as proteins and fats, exhibit varying degrees of molecular motion when exposed to microwave radiation of the same frequency (2450 MHz) and power (5 W) owing to their different dielectric constants. Consequently, different components experience different rates of temperature increase and may require varying durations to reach the same temperature.

**Figure 3:**
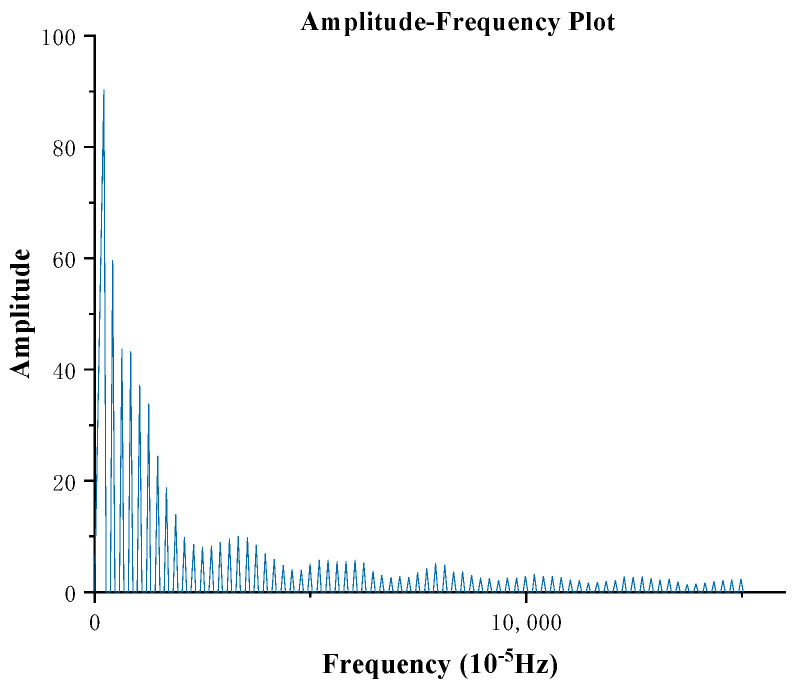
Feature−parameter frequency−domain plot.

Building upon this hypothesis, we analyze the temperature-elevation sequences composed of six feature parameters extracted from ultrasound images using FFT magnitude–frequency analysis (Figure 3). The frequency spectrum represents frequencies corresponding to complete temperature-elevation periods and contains multiple frequency components, indicating the presence of shorter temperature-elevation periods within a longer one. This preliminary analysis helps validate our hypothesis by revealing the underlying temporal patterns and variations in temperature responses captured by the ultrasound image features.

### 2.6. TimesNet

**Figure 4:**
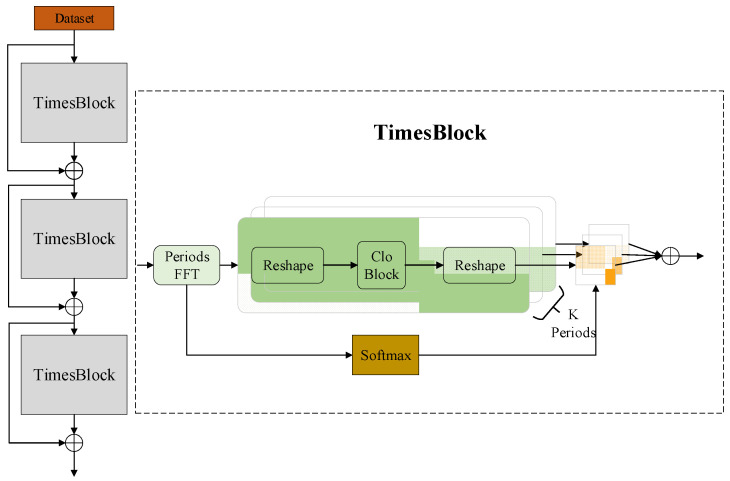
Schematic of the TimesNet structure.

Inspired by TimesNet25 (Figure 4), we decomposed the complex time series containing ultrasound image-feature parameters into transformations within and between multiple periods. This approach involved converting the one-dimensional time series into a set of two-dimensional tensors segmented by periods, extending the temperature information from a one-dimensional sequence to a two-dimensional representation. The multi-period two-dimensional tensors can capture both the long-term periods of complete heating experiments under identical conditions and the shorter heating periods of different components (e.g., protein, fat) within a single heating period. This method enables a more comprehensive representation of temperature dynamics over time, facilitating improved modeling and analysis of heating processes using ultrasound image features.

The one-dimensional sequence was first transformed into a frequency-domain plot using FFT (Figure 3). Thereafter, the normalized amplitudes of multiple feature parameters were individually computed and then averaged to obtain the amplitude of each frequency component, as outlined in Equation (5).

(5) $$A=Avg\left(Amp\left(FFT\left(X_{1D}\right)\right)\right),f_{1},\cdots ,f_{k}=\underset{f_{*}\in 1,\cdots ,\left[\frac{T}{2}\right]}{\arg Topk}\left(A\right),p_{i}=\left\lceil \frac{T}{f_{i}}\right\rceil ,i\in \left\{1,\cdots ,k\right\}$$

Here, $X_{1D}$ denotes the one-dimensional feature-parameter time series, *A* represents the amplitude of each frequency, Amp denotes amplitude computation, Avg is the average calculation, $f_{1},\cdots ,f_{k}$ are the frequencies of the top *K* maximum amplitudes, and $p_{i}$ is the period corresponding to each amplitude.

Subsequently, based on Equation (5), the one-dimensional time series was reshaped using the selected periods and frequencies to form a two-dimensional tensor $X_{2D}^{i}$, where the rows represent the period $p_{i}$ and the columns represent the frequency $f_{i}$, as illustrated in Equation (6).

(6) $$X_{2D}^{i}={\mathrm{Reshape}}_{p_{i},f_{i}}\left(Padding\left(X_{1D}\right)\right),i\in \left\{1,\cdots ,k\right\}$$

This process transforms the one-dimensional ultrasound feature-parameter sequence into a set of two-dimensional tensors containing information about changes within adjacent time points (within a single period) and similar changes across multiple periods. To accommodate the computational capabilities of portable ultrasound devices and reduce parameter count, we employed the lightweight network Cloblock from CloFormer. This allows the simultaneous extraction of information between each heating period and across multiple heating periods, enabling efficient and effective analysis of the temperature dynamics captured by ultrasound image features.

### 2.7. Cloblock

**Figure 5:**
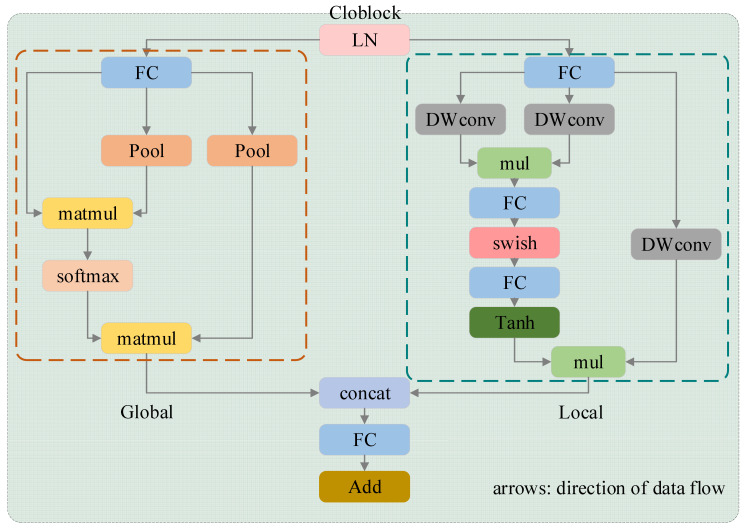
Schematic of the Cloblock structure.

To enhance the information extraction capability of the model and reduce parameter count, we integrated the Cloblock attention-mechanism module into the TimesNet architecture, replacing the inception block. Cloblock features a dual-branch architecture (Figure 5): one branch employs the AttnConv convolution operator with an attention mechanism to capture high-frequency local information of the ultrasound parameter sequence, while the other branch uses a standard attention mechanism to capture low-frequency global information. Cloblock is designed to simultaneously capture global and locally enhanced information, functioning as a lightweight vision transformer. It maintains accuracy while minimizing parameter usage to adapt to the computational constraints of ultrasound devices. This approach enables efficient and effective modeling of ultrasound image features for temperature estimation during MH.

Based on the theoretical hypotheses and research outlined above, we designed an artificial neural network for non-invasive temperature measurement of biological tissues using ultrasound, integrating principles from TimesNet and Cloblock. The data, consisting of ultrasound sequences with extracted feature parameters, were inputted into TimesNet to discover the periodicity in the ultrasound sequences through FFT. This transforms the one-dimensional temporal information into a set of two-dimensional tensors with different frequencies across multiple periods. Subsequently, the lightweight Cloblock module was employed to simultaneously extract local and global features from the two-dimensional tensors. Cloblock leverages attention mechanisms to capture both high-frequency local information and low-frequency global patterns. Finally, the extracted features were converted back into one-dimensional sequences suitable for temperature prediction. These sequences were then fed into the neural network to predict temperature changes based on ultrasound image features. This network architecture enables comprehensive feature extraction from ultrasound data and subsequent temperature estimation, leveraging the FFT-based periodicity analysis of TimesNet and efficient attention-based feature extraction of Cloblock.

### 2.8. Model Parameter Settings

The dataset was divided into training and testing sets at a ratio of 7:3. The loss function employed was the mean squared error (MSE), commonly used for other time-series prediction models. We used the Adam optimizer with an initial learning rate of 0.001 and weight decay of 0.00001, iterating the training process for 1000 epochs. After experimental testing, we found that setting the hyperparameter k periods to five minimized the MSE on the testing set.

## 3. Results

**Figure 6:**
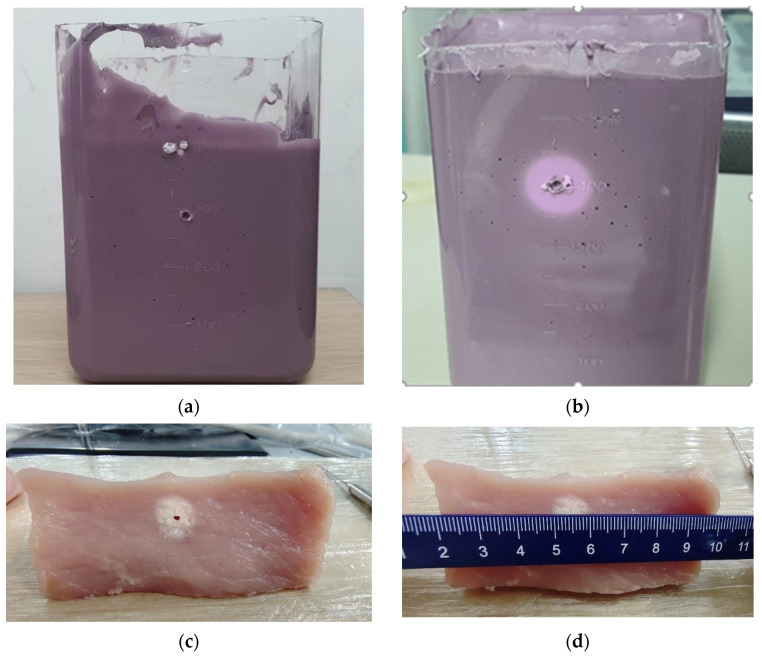
(**a**) Phantom material before heating; (**b**) phantom material after heating; (**c**) ex vivo pork tissue; (**d**) size of the heating area after heating.

After the thermo-ablation experiments, a temperature rise area of approximately 1 cm in diameter was observed in both the phantom and ex vivo pork tissue. In the ex vivo pork tissue, dehydration and protein deformation occurred after microwave radiation (Figure 6a,b). In addition, the color of the phantom changed from purple to pink, as shown in Figure 6c,d.

**Figure 7:**
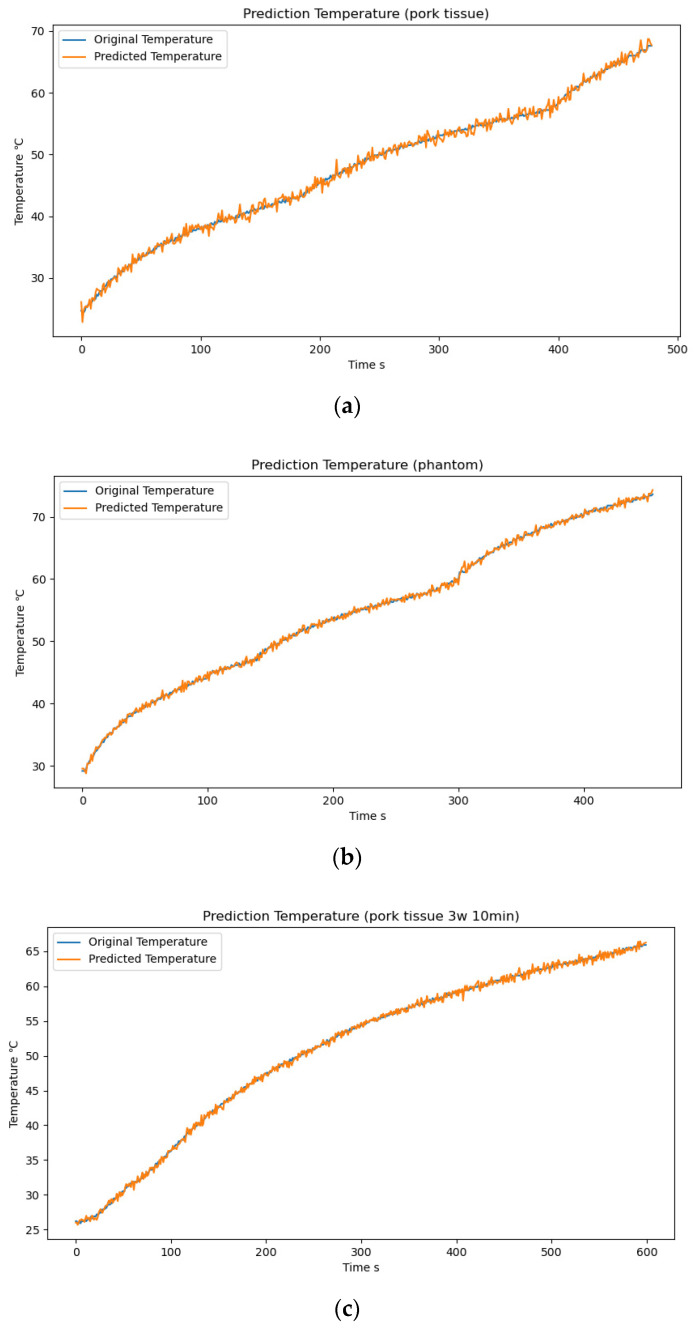

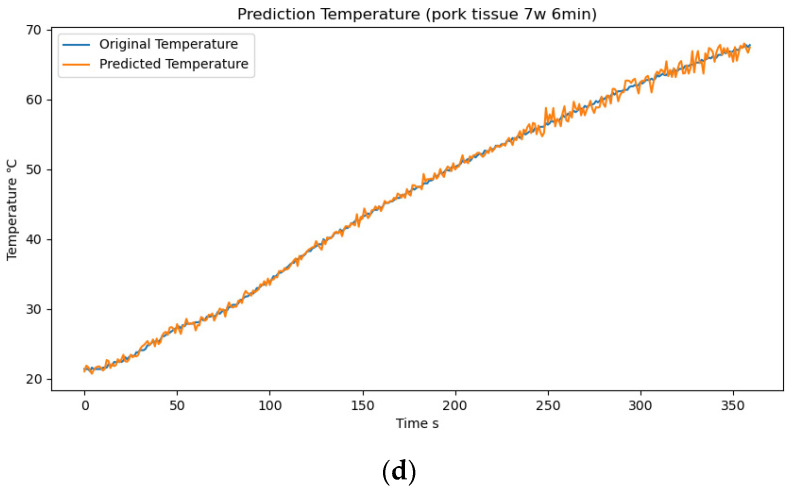
(**a**) Temperature–prediction curve for ex vivo pork tissue; (**b**) temperature–prediction curve for the phantom model; (**c**) temperature–prediction curve for ex vivo pork tissue (3 W/10 min); (**d**) temperature–prediction curve for ex vivo pork tissue (7 W/6 min).

On the ex vivo pork tissue dataset, the neural-network model achieved an average prediction error of 0.706 and an MSE of 0.783; on the phantom dataset, the neural-network model achieved an average prediction error of 0.429 with an MSE of 0.361. For the ex vivo pork tissue, significant temperature errors were observed in the range 25–30 °C (Figure 7a). In the mild thermo-ablation temperature range of 30 °C to 45 °C, the MSE was within 0.5. However, in the thermo-ablation treatment temperature range of 45–65 °C, the MSE gradually increased to around 1.2. Conversely, for the phantom model (Figure 7b), the MSE remained below 0.4 throughout the range of mild hyperthermia (41–45 °C); however, it increased at approximately 60 °C.

To test the prediction accuracy of our neural network on biological tissue samples under varying power levels and heating times, we controlled the same experimental variables while changing only the microwave source power and heating time. We conducted prediction analyses on ten samples of fresh pork heated at 3 W for 10 min and ten samples heated at 7 W for 6 min. The temperature prediction errors were as follows: the mean prediction errors for 3 W/10 min and 7 W/6 min were 0.849 °C and 0.904 °C, respectively. The temperature prediction curves are shown in Figure 7c,d.

## 4. Discussion

**Figure 8:**
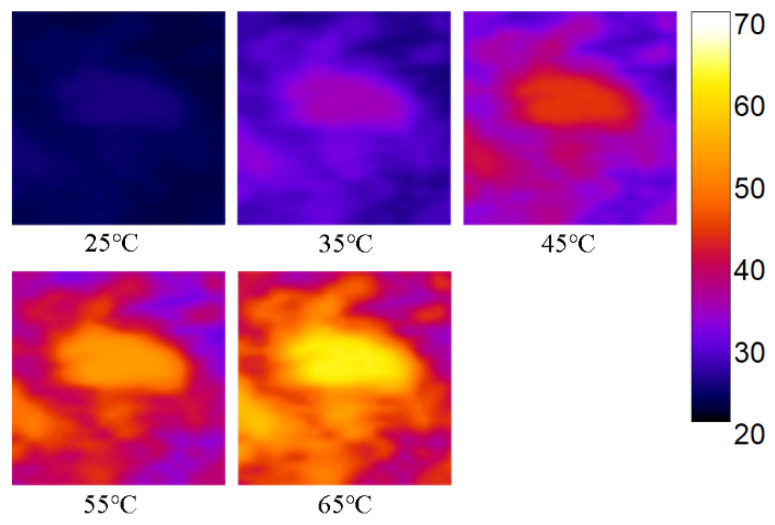
Two-dimensional pseudocolor plot of tissue temperature at 25 °C, 35 °C, 45 °C, 55 °C, and 65 °C.

To visually represent the predicted temperature distribution within the tissue, we created a 2D pseudocolor plot (Figure 8). It can be observed that the heating range closely corresponds to the range of the MH probe.

To assess the temperature-measurement accuracy of our model, we conducted comparisons with polynomial fitting and other common time-series prediction models based on errors and model parameters as summarized in Table 2. The results show that our model outperformed others in terms of temperature-estimation error and model-parameter count.

Discrepancies in temperature prediction between pork tissue and phantom models were attributed to factors such as the presence of fascia and coagulated blood interfering with ultrasound waves within the pork tissue. Additionally, beyond 60 °C, rapid irreversible protein denaturation and cell membrane dissolution further complicated the representation of temperature information through ultrasound image features [35].

The preliminary hypothesis for the varying prediction errors of the model when using different microwave source power levels and heating times for MH on pork tissue is as follows. The 3 W/10 min group demonstrates a relatively stable heating process, allowing the ultrasound image sequence to capture more heating information, resulting in lower prediction errors. Conversely, the 7 W/10 min group exhibits a steeper heating trend in the latter part of the temperature curve (55–65 °C) compared to other power levels (3 W/5 W) owing to the higher power. This leads to tissue dehydration and protein denaturation, causing the ultrasound image sequence to capture insufficient heating information and resulting in higher temperature prediction errors for this group.

This study has several limitations. First, it did not consider tissue deformation during the final stages of heating, which could result in slight displacements of the thermal therapy probe and temperature sensor, leading to errors in the experimental results. Additionally, the heating process of tissues at different initial temperatures was not investigated during the initial stages of the experiment.

Future research will focus on further investigating the various changes occurring within tissues after microwave irradiation and designing heating experiments for a broader range of biological tissues under different initial conditions.

## 5. Conclusions

In this study, we explored the heating principles of tissues under microwave radiation and discovered that ultrasound image parameters during MH can be viewed as a set of sequences with temporal information. We proposed a hypothesis that suggests the presence of short heating periods within each long heating period owing to material composition differences. MH experiments were conducted using ex vivo pork tissue and a phantom model, capturing B-mode ultrasound images along with the corresponding temperature curves. Ultrasound image features were extracted from pre-processed images to establish a dataset, and the hypothesis was preliminarily validated through frequency-domain analysis of the feature-parameter sequences.

Subsequently, an artificial-neural-network model based on TimesNet and Cloblock was designed to model the heating process of ultrasound image features. After multiple rounds of training, a temporal-informed neural network for ultrasound image temperature monitoring was developed. Comparative analysis with polynomial fitting and other time-series prediction models in terms of MSE and parameter count revealed the superiority of the proposed model in temperature-monitoring accuracy and parameter efficiency.

## Figures and Tables

**Table 1:** Six feature parameters along with their Pearson correlation coefficients with temperature.

Feature Parameters	Definition	Pearson Correlation
Average gray level	$\sum_{x=0}^{N-1}i\sum_{y=0}^{M-1}H\left(x,y\right)$	0.895
Gray-level entropy	$-\sum_{x=0}^{N-1}\sum_{y=0}^{M-1}H\left(x,y\right)\cdot \log\sum_{y=0}^{M-1}H\left(x,y\right)$	0.892
Gray-temperature grad	$\frac{T_{t+5}-T_{t}}{{AVGL}_{t+5}-{AVGL}_{t}}$	0.874
Mixture entropy	$-\sum_{x=0}^{N-1}\sum_{y=0}^{M-1}H(x,y)logH(x,y)$	0.825
Inertia	$\sum_{x=0}^{N-1}\sum_{y=0}^{M-1}{\left(x-y\right)}^{2}H\left(x,y\right)$	0.728
Inverse difference moment	$\sum_{x=0}^{N-1}x\sum_{y=0}^{M-1}\frac{H(x,y)}{1+{\left(x-y\right)}^{2}}$	0.726

**Table 2:** Model error and parameter count comparison.

Models/Method	MSE (Pork Tissue/Phantom)	Parameter (MB)
Ours	0.783/0.176	0.046
TimesNet	0.925/0.482	0.067
FEDformer	1.093/0.83	2.604
Autoformer	1.350/1.063	1.428
ConV-LSTM	1.523/1.222	1.259
LSTM	2.672/2.186	0.268
Polynomial curve fitting	3.682/2.864	/

## Data Availability

Data will be made available upon request.
